# An Assessment of Diagnostic Assays and Sample Types in the Detection of an Attenuated Genotype 5 African Swine Fever Virus in European Pigs over a 3-Month Period

**DOI:** 10.3390/pathogens11040404

**Published:** 2022-03-26

**Authors:** Karyn A. Havas, Andrey E. Gogin, Julia V. Basalaeva, Irina P. Sindryakova, Olga L. Kolbasova, Ilya A. Titov, Valentina M. Lyska, Sergey Y. Morgunov, Mikhail E. Vlasov, Timofey A. Sevskikh, Elena Y. Pivova, Dmitry A. Kudrjashov, Kent Doolittle, Silvia Zimmerman, Wendy Witbeck, Luis G. Gimenez-Lirola, Joel Nerem, Gordon D. Spronk, Jeffrey J. Zimmerman, Alexey D. Sereda

**Affiliations:** 1 Pipestone Research, Pipestone Holdings, Pipestone, MN 56164, USA; joel.nerem@pipestone.com (J.N.); gordon.spronk@pipestone.com (G.D.S.); 2 Federal Research Center for Virology and Microbiology (FRCVM), 601125 Volginsky, Russia; agogin@ficvim.ru (A.E.G.); sindryakova.irina@yandex.ru (I.P.S.); olgakolbasova@gmail.com (O.L.K.); titoffia@yandex.ru (I.A.T.); vliska@yandex.ru (V.M.L.); dohliy55555@mail.ru (S.Y.M.); vlasovmikhail1993@yandex.ru (M.E.V.); tsevskikh@ficvim.ru (T.A.S.); lenamail09@inbox.ru (E.Y.P.); dima_kudryashov@mail.ru (D.A.K.); sereda-56@mail.ru (A.D.S.); 3 Swine Division, Cherkizovo LLC, 399870 Lipetsk, Russia; iu.basalaeva@cherkizovo.com; 4 IDEXX Laboratories, Westbrook, ME 04092, USA; kent-doolittle@idexx.com (K.D.); silvia-oliveirazimmerman@idexx.com (S.Z.); wendy-witbeck@idexx.com (W.W.); 5 Innoceleris LLC, Ames, IA 50014, USA; luis.gimenez@innoceleris.us; 6 Department of Veterinary Diagnostic and Production Animal Medicine, College of Veterinary Medicine, Iowa State University, Ames, IA 50011, USA; jjzimm@iastate.edu

**Keywords:** African swine fever, attenuation, diagnostics, detection

## Abstract

African swine fever virus causes hemorrhagic disease in swine. Attenuated strains are reported in Africa, Europe, and Asia. Few studies on the diagnostic detection of attenuated ASF viruses are available. Two groups of pigs were inoculated with an attenuated ASFV. Group 2 was also vaccinated with an attenuated porcine reproductive and respiratory syndrome virus vaccine. Commercially available ELISA, as well as extraction and qPCR assays, were used to detect antibodies in serum and oral fluids (OF) and nucleic acid in buccal swabs, tonsillar scrapings, OF, and blood samples collected over 93 days, respectively. After 12 dpi, serum (88.9% to 90.9%) in Group 1 was significantly better for antibody detection than OF (0.7% to 68.4%). Group 1′s overall qPCR detection was highest in blood (48.7%) and OF (44.2%), with the highest detection in blood (85.2%) from 8 to 21 days post inoculation (dpi) and in OF (83.3%) from 1 to 7 dpi. Group 2′s results were not significantly different from Group 1, but detection rates were lower overall. Early detection of attenuated ASFV variants requires active surveillance in apparently healthy animals and is only reliable at the herd level. Likewise, antibody testing will be needed to prove freedom from disease.

## 1. Introduction

African swine fever (ASF) was first recognized in Kenya in 1921 [1]. The ASF virus (ASFV) is caused by a large DNA arbovirus in the family *Asfarviridae* and genus *Asfivirus* [2] and has at least 24 known genotypes [3]. The different genotypes cause a wide clinical picture in swine species. Disease ranges from subclinical to acute disease [4,5,6]. A virulent genotype II variant of ASFV has caused a panzootic since it was first diagnosed in the Republic of Georgia in 2007 [7,8], and has primarily caused a highly virulent and deadly form of hemorrhagic disease in infected pigs, with only 2–10% of pigs surviving infection [9]. This variant is now found across Eastern Europe, Asia, and the Caribbean [8]. Diagnostics need to be used with the characteristics of the circulating variants in mind. Highly sensitive real-time polymerase chain reaction (qPCR) assays are needed for early detection of acutely infectious virulent ASFV, as well as for subacute infections that have weak viremias. Antibody detection is critical for detection of animals that survive as well as for low virulent strains [10].

Since the beginning of the panzootic, some European countries and China progressed from reporting primarily acute cases associated with the panzootic strain to also reporting mild and chronic cases, suggesting viral attenuation [9,11,12,13]. Historically, chronic disease in epizootic strains outside of Africa have been associated with natural reversion and the use of attenuated vaccines that reverted to low virulence [14]. China reported chronic disease in sows when pigs developed severe issues with fertility and tested positive for ASF despite never showing signs of acute illness. This was associated either with the black market use of unapproved vaccine strains, as reported by Reuters News Service [15], or natural attenuation of the circulating virus [13].

In the United States, samples that can be used for diagnostic testing via qPCR include blood, serum, or tonsil scrapings for ante-mortem samples, and tonsil, spleen and/or lymph nodes for post-mortem samples [16]. This makes on-farm surveillance for continuity of business or early detection difficult for commercial farms. For routine endemic disease surveillance and diagnostics, oral fluid samples are the easiest to collect from large groups of pigs and are widely used in the U.S. swine industry [17,18]. There are many National Animal Health Laboratory Network member laboratories that routinely run oral fluids for endemic disease diagnosis and detection according to their website information. Oral fluid samples can be used to detect antibodies and pathogen nucleic acid for multiple diseases, including porcine reproductive and respiratory syndrome virus (PRRSV) swine influenza, and others [17,19,20,21]. Furthermore, oral fluid samples were useful to detect ASFV nucleic acid in infected pigs prior to clinical signs in experimental settings using acute viral strains [22], and can detect ASFV in a pen of 20 animals when only one animal is infected with an acute viral strain in experimental settings [23]. Detection of antibodies against ASFV is also possible using oral fluids [24,25]. Beemer et al. [26] estimate that the use of oral fluids could reduce sample sizes and thus resource needs by 40% during an outbreak as well. There is a need and potential for use of this sample type in early detection on farms for ante-mortem detection of ASF in infected pigs, but there is no work published on the detection of attenuated strains using oral fluid samples.

When considering attenuated ASFV, the temporal dynamics of viral nucleic acid and antibody detection between ante-mortem sample types is not well-described. A study evaluating antibody and nucleic acid detection over time using the highly virulent Georgia 2007 genotype II ASFV variant was done but was limited in demonstrating long term dynamics due to the high mortality [27]. For example, in the study of the 2007 genotype II variant, the researchers were only able to detect one pig with antibodies before death. Other studies used moderately virulent ASFV strains, and showed detection of viral nucleic acid in blood for up to 70 days [28] and 91 days [29]. Another study published in 2021 compared virulent, moderately virulent and attenuated strains from Europe over a 76-day period using oral pharyngeal swabs, whole blood and fecal swabs [30], but did not evaluate oral fluids or differences between diagnostic assays. A longer-term study that evaluates other attenuated strains, more diagnostic sample types and a variety of commercially available assays is needed to understand the temporal dynamics of antibody shedding compared to nucleic acid detection in attenuated viruses.

ASF is clinically indistinguishable from other endemic and ubiquitous diseases of swine, such as porcine reproductive and respiratory syndrome virus (PRRSV). Modified live vaccinations are widely used to reduce the spread of PRRSV in commercial swine systems and, nonetheless, outbreaks of this disease are common [31]. It is not understood how diagnosis of ASFV by antibody or nucleic acid detection is affected by such a co-infection either.

The purpose of this study was to evaluate the dynamics of ASF viral nucleic acid and antibody detection across different commercially available diagnostic assays. The evaluation occurred in pigs inoculated with an attenuated strain of a previously virulent variant (Group 1) and in pigs vaccinated for PRRSV just prior to infection with ASFV (Group 2).

## 2. Results

### 2.1. Clinical Presentation and PRRS Testing Results

Only one animal in Group 1 developed a temperature of ≥40 °C in the 10 days post inoculation (dpi) with the ASFV MK-200 attenuated variant. In Group 2, all animals showed an increased temperature from their day 0 measurement by three days post-inoculation (dpi). Four animals in Group 2 had a temperature of greater than 40 °C for one to two days. No other clinical signs were detected in the inoculated animals.

PRRSV nucleic acid was detected in four pigs in Group 2 and in two of the three control pigs on Day 10 in blood samples. One pig in Group 2 also had a positive result on oral fluids using qPCR.

### 2.2. Extraction and Real Time Polymerase Chain Reaction

Inhibition was evaluated based on extraction and amplification control cycle threshold (Ct) values and by the shape of the qPCR amplification curves. Overall, less than 1.9% of samples were invalid. This statistic does not include oral fluids samples that were discarded due to mechanical issues during extraction on one assay and extraction failures on another. They would have been tested using the IDEXX RealPCR^TM^ ASFV DNA test (IDEXX Laboratories, Westbrook, ME, USA) (IDEXX qPCR). This included a loss of 35 oral fluid samples from Group 1, 48 from Group 2 and 9 from the control group for days 36 to 50 for each extraction method. There was not enough remaining sample to re-run the extractions from the beginning. The percent of blood samples that were invalid was significantly higher for the IDEXX RealPCR^TM^ DNA/RNA magnetic bead extraction assay (RealPCR^TM^) (IDEXX Laboratories, Westbrook, ME, USA) (RealPCR^TM^) and the MagMax Core DNA/RNA extraction assay (Thermofisher Waltham, MA, USA) (MagMax) when tested with the ID Gene^®^ ASF Triplex qPCR assay (Innovative Diagnostics, Grabels, France) (IDVet qPCR), which uses both an endogenous and exogenous internal control, 7.8% and 12.5% respectively, otherwise no significant differences were noted (See Table 1).

The MagMax extraction with the IDEXX qPCR detected ASFV DNA in blood in two out of three pigs on 2 dpi, in the oral fluid samples of all three pigs on 1 dpi, and in one of three buccal swabs and tonsillar scrapings on 1 dpi. The MagMax extraction and IDVet qPCR categorized one blood sample out of three as positive on 2 dpi, two out of three oral fluids samples as positive on 2 dpi, and one buccal swab and one tonsillar scraping sample as positive on 1 dpi. These were the earliest time points of detection.

The positivity rate among valid samples was evaluated for each group, sample type, extraction assay and qPCR assay to provide a thorough comparison of detection rates in inoculated animals across sample types and over time periods (Table 2). Overall, for Group 1, there was no combination of extraction and qPCR kits that returned a significantly higher positivity rate over time and sample type. Based on the highest positivity rate alone, the MagMax extraction with the IDEXX qPCR gave the highest values for all sample types in the overall category. Blood had a significantly higher positivity rate when compared to buccal swabs and tonsillar scrapings in the overall category and between some of the time periods. Blood also had the highest positivity rate among all sample types from 8–21 dpi (85.2%) when using the RealPCR^TM^ extraction and IDVet qPCR assays to test the 27 samples. This was not significantly different from any of the other diagnostic assays for blood from 8–21 dpi. Oral fluids had a higher positivity rate compared to buccal swabs and tonsillar scraping in samples collected 1–7 dpi. Oral fluids had the highest positivity rate among all the sample types from 1–7 dpi with the MagMax extraction and the IDEXX qPCR assay returning positive results for 83.3% of the 18 samples tested. The positivity rate of samples dropped over time across all assays and sample types. During the last time period from 64 to 93 dpi, oral fluids extracted with the MagMax assay and tested with a IDEXX qPCR assay had the highest positivity rate (40.6%, n = 32).

Group 2 had a consistently lower positivity rate than Group 1 across all sample types, assays, and time periods, but there was not a significant difference. Many of the other trends discussed in Group 1′s sample results were replicated. Blood was again significantly different from buccal swabs and tonsillar scrapes overall and between time periods based on diagnostic assays used, but it was not significantly different from oral fluids. The highest positivity rate (60.7%) was found in blood samples taken 1–7 dpi when using the RealPCR^TM^ extraction and IDEXX qPCR assays, but there was not a significant difference across diagnostic assays. The highest oral fluid positivity rate (55.6%, n = 9) was found in samples collected from 22 to 42 dpi that were extracted with RealPCR^TM^ and tested with the IDEXX qPCR assays; this did not significantly differ across diagnostic assays. This was also the period where data was lost due to failed extractions. Again, detection dropped as time passed. In the samples collected from 64 to 93 dpi, the highest positivity rate (45.5%) was found with oral fluids extracted using the MagMax and tested on IDEXX qPCR.

Among all the known negative samples, two oral fluid sample from two animals in the control group had a positive result when using the RealPCR^TM^ extraction and IDVet qPCR. This occurred for samples from 20 and 30 dpi that were tested on the same plate. The Ct values were 37.5 and 38.5.

Group 1 was inoculated with an attenuated genotype 5 African swine fever virus variant and Group 2 was inoculated with the ASF variant 10 days after being inoculated with a modified live European strain porcine reproductive and respiratory syndrome virus vaccine.

Table 3 summarized the correlation between the Ct values and the agreement between qPCR assays based on extraction assay and sample type. There was moderate to substantial agreement between the qPCR assays in Group 1 and 2 for both extractions across all samples. Despite having diagnostic agreement, the Ct values for Group 1′s oral fluid samples exhibited no correlation among Ct values between qPCR assays on both extractions. Although the Ct values were not statistically different between the qPCR assays, the IDVet qPCR assay had consistently lower median Ct values across extraction kits and sample types.

### 2.3. Enzyme Linked Immunosorbent Assay

Table 4 summarized the positivity rate for antibody detection using the various diagnostic assays starting at 7, 12 and 17 dpi. Innoceleris (Ames, IA, USA) provided a fewer number of reactions, so fewer samples were tested and there was no sample size difference between the cohorts of samples from 12–93 dpi and 17–93 dpi in Group 1 and 7–93 dpi and 12–93 dpi in Group 2. The earliest detection of antibodies against the ASFV MK–200 variant was in serum from one pig on day nine (Group 1). Group 2 had five pigs with detectable antibodies in serum between 6 and 9 dpi. One also had detectable antibodies in oral fluids. The first pig had detectable antibodies in serum and oral fluids on 6 dpi (Group 2). Consistent detection of antibodies began on 12 dpi in serum and on 16 dpi (Group 2) or 17 dpi (Group 1) for oral fluid samples. Antibody detection increased over time, but there was not a significant difference between time points in either group or sample type. Group 2 results had a consistently lower positivity rate than Group 1, despite the earlier detection, but there was not a significant difference between the groups.

There was a significant difference in detection of antibodies in oral fluids versus serum across all assays in Group 1 and in the Innoceleris and Ingenasa (Madrid, Spain) assays in Group 2. The best antibody detection in serum was with the Ingenasa ELISA in Group 1 at 17 dpi (91.1%), followed by Innoceleris at 90.9% and ID Screen^®^ competition ELISA at 90.4%. There was no significant difference between the assays. The best antibody detection in oral fluids was with the ID Screen^®^ indirect oral fluids ELISA after 12 dpi (68.4%), followed by the Innoceleris (51.1%), and the Ingenasa (0.7%). There was no significant difference between the Innoceleris and ID Screen^®^ assays (Innovative Diagnostics, Grabels, France). The Ingenasa ELISA had the poorest performance on oral fluids, but also did not have a protocol in the kit insert for this sample type. As for false positives on samples from the control group, the Ingenasa ELISA had seven positive serum samples from one animal in Group 4, but all other control samples were negative. This animal was positive on the Ingenasa ELISA at all samplings after 20 dpi. This was a different animal than was positive by qPCR. Finally, antibodies in serum from 93 dpi were detected in all animals using the IDVet and Innoceleris ELISA assays and in all but one animal’s serum when using the Ingenasa ELISA assay. Antibodies in oral fluids were detected at 93 dpi in one animal sample by the Innoceleris ELISA assay, in three animal samples by the IDVet ELISA assay, and in none of the samples when using the Ingenasa ELISA assay.

Table 5 summarizes the agreement between assays. The serum had substantial agreement in Group 1 and 2, while oral fluids had fair agreement in Group 1 and 2. The ID Screen^®^ competitive ELISA for serum and the Innoceleris ELISA had almost perfect agreement for serum in Groups 1 and 2. The ID Screen^®^ indirect ELISA and the Innoceleris ELISA had substantial agreement for oral fluids in Groups 1 and 2. The Ingenasa ELISA had substantial agreement to the other assays in Group 1 and with Innoceleris in Group 2 and moderate agreement to the ID Screen when evaluating serum, but had poor, slight to fair agreement for all other comparisons.

## 3. Discussion

Attenuated ASFVs have been developed or identified as part of the vaccine discovery [14,32,33], and are emerging in Asia [12,13] and Europe [9,34], and are present in East Africa [6,35,36]. This study evaluated the detection of an attenuated virus variant developed as part of vaccine discovery in groups of pigs, one of which was also vaccinated using a modified live European PRRSV vaccine. There were limitations to this study that must be considered. First, pig group sizes were limited due to space and cost constraints associated with working in a BSL-3Ag space. This limited the ability to detect statistical differences. One control group (ASFV and PRRSV MLV negative) was accidentally infected with a virulent ASFV strain that was part of a separate study, and those results were discarded. Although having an additional control would have been valuable, we did maintain an adequate control in this study (ASFV negative and PRRSV MLV positive). The Ingenasa INgezim blocking ELISA’s protocol only mentions the use of serum, but as one of the most widely used ASF antibody ELISAs [34], it was included in this study and evaluated on oral fluids, despite not having a protocol for oral fluids.

There were some significant differences across assay types and combinations, but there was no consistent combination of extraction and qPCR kits that returned a significantly higher positivity rate over time and sample type. Using the measure of highest positivity rate for the overall testing, the MagMax extraction with the IDEXX qPCR gave the highest values in Group 1, and the IDEXX qPCR with both extractions gave the highest positivity rates overall in Group 2. However, there was variation between time periods and sample types on what would yield the highest positivity rate. For example, for Group 1′s blood samples from day 8–21 dpi, it was the RealPCR^TM^ extraction and IDVet qPCR and for day 8–21 oral fluid samples it was the MagMax extraction and IDVet qPCR. Laboratories should fully characterize the qPCR assays they use to optimize Ct cut-off values, maximum number of thermocycles to allow, and to set Ct limits for extraction and amplification controls for the sample type of choice. Further work on sample validation should be done to better characterize sensitivity and specificity on antemortem sample types for attenuated viruses as well.

There was a significant difference in sample inhibition, the IDVet qPCR had a higher rate of invalid blood samples compared to IDEXX. Samples that have a high rate of inhibition (>1%) may require the addition on internal controls to detect false negatives (Buckwalter et al., 2014). In this study, all sample types had an inhibition rate of >1% with some combination of extraction kits and qPCR assays. qPCR inhibition can be measured in two ways: with endogenous and exogenous internal controls. Exogenous controls are added to the sample prior to extraction to measure inhibition. Endogenous controls are inherently present in the sample, and the amount present can vary based on the sample type and sample handling. Thus the inhibition measured is not as controlled with endogenous controls and its reliability differs based on the cut-off value [37,38]. Furthermore, if small amounts of virus nucleic acid are present, the endogenous control can outcompete the ASFV gene target and result in a false negative [38]. The IDEXX and IDVet qPCR kits used two different internal control systems. The IDVet qPCR used an exogenous and endogenous internal control and the IDEXX kit used an endogenous control. Blood inhibition significantly differed between the qPCR kits with the IDVet kit having a higher rate of invalid samples (7.8% with the IDEXX extraction and 12.5% with the MagMax extraction). For the IDVet kit, it was the exogenous internal controls that invalidated all but four of the invalid blood samples. In addition, all ASF viral targets for those invalidated samples yielded negative results. The invalidation may have prevented having false negatives. In comparison, the IDEXX results for the same samples classified 15 as negative, four as positive, and one was invalid. If there were low viral titers in the samples used, exogenous controls may be a more reliable way to identify potential inhibition due to the competition that may occur between the endogenous control and the target ASFV gene. The RealPCR^TM^ extraction also had one plate that was discarded due to a high level of endogenous control failures, although the reason for this is less clear. Further work is needed on ante-mortem sample types to ensure reliable antibody and nucleic acid detection across different sample types.

The ability to detect the nucleic acid of attenuated viruses was curtailed in this study compared to previous studies that evaluated a wide range of ASFV collected from field isolates. The detection of ASFV with qPCR has historically led to a high sensitivity [34,39,40,41,42]. However, our findings when trying to detect nucleic acid from an attenuated virus were highly variable across sample types. In Group 1, blood samples had an overall positivity rate in the mid–40s and were lower in Group 2. At best, the positivity rate for blood samples in Group 1 was 85.2% from 8–21 dpi and was 60.7% for Group 2 during the same period. There was moderate to substantial agreement across tests as well, so classification was consistent across the different assays, suggesting that this may not be a diagnostic detection issue as much as an infection dynamic. This suggests a lower level of viremia or intermittent viremias and the shedding involved with such viruses. These results align with previous work. Studies done using moderately virulent viruses showed greater than 50% detection in blood up to 70 and 91 dpi [28,29]. Sporadic shedding and detection of an attenuated virus over time was also found by one other study in pigs inoculated with a non-hemadsorbing Latvian ASFV variant [30]. This makes early detection difficult, and more work is needed to understand the dynamics of viral persistence and shedding with attenuated ASFV viruses.

Surveillance for early detection relies on detection of viral nucleic acid or other antigens shortly after infection. To achieve this with attenuated viruses will require regular and on-going testing in apparently uninfected herds, or active surveillance. Increasing the testing for ASFV in areas where it has become endemic or in areas where it is a high threat is the only way to ensure detection of these low virulent strains. For increased surveillance system sensitivity, the herd rather than the animal will need to be the epidemiological unit, since animal-level testing is inconsistent regardless of sample type, and repeat testing is needed to reliably detect the nucleic acid in an animal. A sampling frequency should be determined to return a reliable level of sensitivity in the surveillance system for a given sample type despite the lower diagnostic sensitivity. Oral fluid samples may be the most appropriate to detect attenuated viruses, as they can be collected repeatedly with no negative welfare impact on the animals, are less likely to contribute to disease spread, and are less taxing on manpower resources, although collecting individual oral fluid samples may only be possible in farrow-to-wean facilities that use crates in the gestation barns. Furthermore, evaluation of oral fluids as a pen-level aggregate sample type for attenuated virus detection is needed to allow this sample to be more widely used in production facilities that house pigs in pens. The bleeding of animals may spread disease, as animals must be individually snared and physically handled to be bled, leading to equipment and humans spreading disease within a barn. There is also the potential that any blood that contaminates the environment will further spread disease. Pooling of samples may further reduce detectability, although this was not evaluated in this study. Additional work is clearly needed on the diagnostic capabilities to best develop acceptably sensitive surveillance programs.

The antibody ELISA assays’ abilities to detect positive animals were more reliable than nucleic acid detection, with an attenuated ASFV overall, particularly after 12 dpi, but detection was possible as early as 6 dpi. The positivity rate using serum samples was over 90% in Group 1 and over 80% in Group 2, and was lower in oral fluid samples. Surveillance for freedom of disease can make use of antibody detection, and, overall, this may provide a more reliable and economical method to prove freedom from ASFV attenuated viruses. The weakness is that it delays diagnosis by up to two weeks in a system under surveillance. This is still better than potentially many months should a swine system forego use of active surveillance. There are similar considerations to active surveillance for viral nucleic acid. The herd should be the epidemiologic unit and, depending upon the testing frequency needed to meet surveillance system sensitivity criterion, oral fluids should be considered. Therefore, ELISA assays used should be evaluated against oral fluids to be sure they provide reasonable detection; aggregate oral fluids samples should be evaluated to be sure detection is reliable at the pen-level, and pooling of serum and oral fluids samples should also be evaluated. The increased consistent reliability of antibody testing is useful when paired with stringent biosecurity protocols to help prove freedom of disease for movement when animals have been at a site for more than 14 days under rigorous biosecurity protocols.

Additional work is needed to understand how to detect attenuated ASFVs. This study evaluated a single attenuated virus, and there may be differences in detection across diagnostic assays for different viruses. Furthermore, the consistent trend of a lesser rate of detection in the PRRSV vaccinated group is concerning and should be repeated in other studies with other attenuated viruses to determine if this is an anomaly in this trial, or a unique component of coinfection. Validation of aggregate oral fluid samples and pooled samples using attenuated viruses should also be done. Finally, further work on the transmissibility of attenuated viruses is needed to understand the contribution these viruses have in propagating disease. Previous work on moderately virulent viruses determined transmission up to 91 days after infection did not occur despite viral nucleic acid detection [29], but this should be verified with attenuated viruses.

## 4. Materials and Methods

### 4.1. Pig Source and Grouping

The study used 22 pigs that were 30–35 kg and approximately 12–weeks old and provided by a local commercial swine production company from a PRRSV negative site. They were housed in a BSL3-Ag laboratory that was part of the Russian Federal Research Center of Virology and Microbiology (FRCVIM) in the Vladimir region of Russia. There was a six-day acclimation period prior to the start of the study. During the acclimation period, pigs were exposed to ropes for chewing.

The pigs were randomly allocated into four groups and housed in four separate rooms. Groups 1 and 2 had eight pigs, six were individually housed and two were housed together. Groups 3 and 4 were control groups, had three pigs each, and were all individually housed. Group 1 was inoculated with an ASFV MK-200, an attenuated strain, on day 0, Group 2 was inoculated with a PRRSV European subtype (*Betaarterivirus suid 1*) modified live vaccine (MLV) on day 0 and the ASFV MK-200 strain on day 10, Group 3 was not inoculated with anything, and Group 4 was inoculated with the PRRSV vaccine on day 0.

### 4.2. Inoculations

In this study, we used an attenuated ASFV hemadsorbing variant (MK–200) characterized by a prolonged viremia post-inoculation, and derived from the highly virulent Mozambique 1978 strain (genotype 5 and seroimmunotype 3 virus) [43,44]. Its preparation and clinical presentation in inoculated animals have been previously described [44]. Briefly, determination of infectious activity in the viral suspension was done by conducting a two-day titration in a primary porcine macrophages culture. Viral titers were then calculated using the Kerber method with the Ashmarin-Vorobyov modification [45].

Pigs were inoculated with 10^6.0^ HAU_50_ intramuscularly in the trapezius muscle. Temperatures were monitored for the first week to evaluate the virulence of the infection. A PRRSV modified live vaccine, Porcilis^®^, produced by Intervet in the Netherlands, was injected intramuscularly in the neck behind the ear into Group 2 pigs as per the manufacturer’s instructions.

### 4.3. Sample Collection

Individual animal samples were collected throughout the 93-day period of this study. For Groups 1 and 2, the first nine days post-ASF inoculation samples were collected daily from sub-sets of two to three pigs. Each pig was sampled every three days. This was done because of animal welfare concerns about daily venipunctures and tonsillar scrapings. From days 10 to 60, pigs were sampled every three to four days, and then weekly from days 60 to 93. For Groups 3 and 4, pigs were sampled every ten days throughout the study period. Tonsillar scrapings, buccal swabs, oral fluids, serum, and whole blood were collected. To collect oral fluids, cotton tri-stranded ropes (TD PROMT LLC, Lipetsk, Russia) were hung at shoulder height for 20–30 min in front of each animal. The housed animals were separated during this period. The pigs chewed the ropes, and the oral fluids were collected in a plastic bag (TD PROMT LLC, Lipetsk, Russia) and then a plastic vial (Fisher Scientific Company LLC, Pittsburgh, PA, USA). They were centrifuged at 1200× *g* for 2 min and the supernatant was collected. Serum and whole blood were collected using jugular venipuncture. Whole blood was collected into EDTA tubes (TD VIK LLC, Lyubertsy, Russia). Serum was collected into coagulation tubes (TD VIK LLC, Lyubertsy, Russia), allowed to clot, and then centrifuged for 10 min at 2000× *g* and the serum collected. Tonsillar scrapings were collected using a long-handled spoon, snare and/or speculum to gently scrape the tonsil in a back-to-front direction to collect at least a spoon full of exudate and added to a collection vial with 3 mL of viral transport media (TD PROMT LLC, Lipetsk, Russia). Buccal swabs were collected by taking a polyester swab on a plastic shaft and running it in between the teeth and cheek from the commissure of the lips to the angle of the jaw until it was soaked. It was then placed in 3 mL of viral transport media (TD PROMT LLC, Lipetsk, Russia). Three aliquots were made of all the samples described above and stored at −40 °C until testing in 5 mL cryovials (Simport Scientific, Quebec City, QC, Canada) or 2 mL cryovials (Corning Inc, Corning, NY, USA). All equipment was cleaned and disinfected between pigs and uses. All samples went through one freeze thaw cycle prior to testing. Group 3 was accidentally infected with a highly virulent ASF strain during the experiment, and those results were discarded.

### 4.4. Pain Management and Euthanasia

Pigs were given 4.4 mg/kg of flexprofen (TD VIK LLC, Lyubertsy, Russia), a non-steroidal anti-inflammatory drug, for pain management as needed. Final euthanasia was performed using a captive bolt device. This study was approved by the Pipestone Research Institutional Animal Care and Use Committee (Protocol # 2021-5; 26 January 2021).

### 4.5. Diagnostics

Serum and oral fluid samples were tested for antibodies against ASFV. Three different companies’ assays were used: Innovative Diagnostics (IDVet; Grabels, France), Ingenasa (Madrid, Spain), and Innoceleris (Ames, IA, USA). IDVet provided two assays, the ID Screen^®^ African Swine Fever Oral Fluids indirect ELISA, which uses VP32 (also known as VP30), VP62, and VP72 recombinant proteins [46], and the ID Screen^®^ African Swine Fever Competition ELISA, which uses VP32 proteins, for use with serum [47]. Innoceleris provided an indirect ELISA, which detects IgG antibodies against ASFV, with separate instructions for analyzing serum and oral fluids [48,49]. The Ingenasa’s INgezim PPA COMPAC blocking ELISA was purchased for this study; it uses a monoclonal antibody against the ASFV VP72 protein, to use on serum and oral fluids [50]. All assays were performed following manufacturer’s instructions.

Oral fluids, whole blood, tonsil scrapings, and buccal swabs were tested for ASFV nucleic acid. Extraction was done using two commercially available assays: the MagMax Core DNA/RNA extraction assay (Thermofisher Waltham, MA, USA) was purchased, and the RealPCR^TM^ DNA/RNA magnetic bead assay (IDEXX Laboratories, Westbrook, ME, USA) was donated. Extractions were performed using a KingFisher Flex (Thermofisher Scientific, Waltham, MA, USA) as per manufacturer’s instructions. Viral DNA amplification was done using two donated commercial qPCR assays: the IDEXX RealPCR^TM^ ASFV DNA test and the ID Gene^®^ ASF Triplex. IDEXX was used as an endogenous internal extraction control and IDVet was used as an endogenous and exogenous internal extraction control. The exogenous control was added to the lysis buffer prior to extraction. All assays were performed following manufacturers’ instructions on a CFX-96 (BioRad Laboratories, Hercules, CA, USA). When the Ct cut-offs were visually evaluated, if the baselines of the amplification curves crossed the automatically set up threshold, the threshold was set to 5% of the value of the plateau of the positive amplification control, and if the baseline crossed the 5% threshold, the 10% cut-off was used [51].

PRRSV was tested for on samples from the vaccinated pigs in Group 2 and the controls. Blood and oral fluid samples were extracted using the MagMax assay and tested using an Amplisense PRRS reverse transcriptase qPCR assay (Helikon Company LLC, Moscow, Russia). Samples were tested for PRRSV every ten days in the control animals and every 10 to 14 days in Group 2 pigs.

### 4.6. Validation of Diagnostic Assay Results

Positive results on the antibody ELISA assays were valid if they met the manufacturer’s instructions for a positive result and the negative control was negative. For negative results, the results were valid if they met manufacturers’ instructions for a negative result and if the positive control was positive.

For the nucleic acid detection results, positive results were considered valid if the negative extraction and amplification controls did not have a Ct value, the amplification curves were normal, and if the samples’ Ct value was >15 and <40. Negative results were considered negative if the positive extraction and the positive amplification controls were positive as per the manufacturers’ instructions, and if the sample had no Ct value, or the Ct value ≤15, or ≤40. Data was entered into spreadsheets using Microsoft Excel version 16.55 (Microsoft Corporation, Redwood, WA, USA).

### 4.7. Statistical Analysis

For nucleic acid detection, the results were summarized by pig group, extraction assay, qPCR assay, and sample type for the following time periods: 1 to 7 dpi with the ASF MK-200 variant, 8 to 21 dpi, 22 to 42 dpi, 43 to 63 dpi, and 64–93 dpi. For antibody ELISA results, data was summarized by pig group, assay, 7–93 dpi, 12–93 dpi, and dpi 16– or 17–93. Frequency and proportions were calculated with 95% confidence intervals when appropriate. For sample sizes less than 50, the Wilson method was used to calculate proportional confidence intervals and the Agresti-Coull method was used for sample sizes greater than 50 [52]. Overlapping confidence intervals were used to determine insignificant differences between groups. Agreement between diagnostic assays was evaluated using a prevalence and bias weighted Kappa statistic [53,54]. Agreement was poor (Kappa statistic < 0), slight (0–0.2), Fair (0.21–0.4), moderate (0.41–0.6), substantial (0.61–0.8), and almost perfect (0.81–1) [55]. For qPCR results, the correlation of Ct values across qPCR assays and extraction types was also calculated. Finally, known negative samples were used to evaluate the ability to classify a negative by all assays, but there were not enough samples to appropriately call this specificity. Known negatives were the samples collected on the day of inoculation prior to inoculation with ASFV (day 0 for Group 1 and day 10 for Group 2). Group 2 and Group 4 were vaccinated with the PRRS MLV on day 0 and had baseline samples collected that day as well. All analysis was performed in Stata version 16.1 IC (Stata Corporation, College Station, TX, USA).

## 5. Conclusions

Attenuated strains of ASFV are present in Europe, Asia, and Africa, and the diagnostic detection of infected animals is poorly studied. This study determined that pigs infected with attenuated viruses are most reliably detected using antibody detection, but the detection is delayed for 12 days. Detection of viral nucleic acid is possible, but it is not consistent in antemortem samples at the individual animal level. Therefore, the epidemiologic unit for attenuated ASFV strains and antibodies against attenuated strains must be the herd. Surveillance and monitoring system design must allow for repeat testing to achieve reasonable surveillance sensitivity in apparently healthy or mildly ill animals. Oral fluids are an ideal sample type for antibody and nucleic acid detection when considering manpower and animal welfare, but further work is needed to evaluate its impact on diagnostic sensitivity when trying to detect attenuated ASFV at the pen-level or when pooled to adequately determine testing frequency.

## Figures and Tables

**Table 1:** The percent of sample inhibition when testing for African swine fever by sample type, polymerase chain reaction assay, and extraction assay.

	Blood		Oral Fluid		Buccal Swab		Tonsillar Scraping	
	# Tested	% Invalid (95% CI)	# Tested	% Invalid (95% CI)	# Tested	% Invalid (95% CI)	# Tested	% Invalid (95% CI)
**IDEXX Extraction**								
IDEXX PCR	373	0.3 (0, 1.7)	281 *	3.6 (1.9, 6.5)	373	0.3 (0, 1.7)	373	0.8 (0.2, 2.5)
IDVet PCR	373	7.8 (5.4, 11)	373	0.8 (0.2, 2.5)	373	0.3 (0, 1.7)	373	0.8 (0.2, 2.5)
**MagMax Extraction**								
IDEXX PCR	373	1.1 (0.3, 2.8)	281 *	2.1 (0.9, 4.7)	373	1.1 (0.3, 2.8)	373	2.1 (1, 4.2)
IDVet PCR	375	12.5 (9.5, 16.3)	371	1.9 (0.8, 3.9)	372	0.3 (0, 1.7)	374	0.3 (0, 1.7)

95% CI is 95% confidence interval. * A MagMax plate was extracted twice due to a power outage and 92 oral fluid samples were excluded. The IDEXX extraction plate controls failed, and 92 oral fluid samples were excluded. These were excluded from this analysis.

**Table 2:** Percent of positive samples for nucleic acid detection of an attenuated African swine fever stratified by diagnostic assays, sample types, group inoculated, and time period.

*PCR Kit*	IDEXX		Innovative Diagnostics	
*Extraction Kit*	MagMax	IDEXX	MagMax	IDEXX
**GROUP 1**	% (95% Confidence Interval), n		% (95% Confidence Interval), n	
Blood	48.7 (41.1, 56.5), n = 158 ^a^	45.9 (38.4, 53.7), n = 159 ^a^	46.4 (38.3, 54.7), n = 138 ^a^	44.2 (36.2, 52.5), n = 138 ^a^
0 < dpi ≤ 7	52.6 (31.7, 72.7), n = 19	36.8 (19.1, 59), n = 19 ^a^	30 (14.5, 51.9), n = 20 ^b^	33.3 (15.2, 58.3), n = 15
7 < dpi ≤ 21	62.1 (44, 77.3), n = 29	82.1 (64.4, 92.1), n = 28 ^a^	71.4 (52.9, 84.7), n = 28 ^a^	85.2 (67.5, 94.1), n = 27 ^a^
21 < dpi ≤ 42	64.6 (50.4, 76.6), n = 48	50 (36.4, 63.6), n = 48	53.5 (38.9, 67.5), n = 43	60 (44.6, 73.7), n = 40 ^a^
42 < dpi ≤ 63	31.3 (18, 48.6), n = 32	37.5 (22.9, 54.7), n = 32	37.5 (21.1, 57.3), n = 24 ^a^	24.1 (12.2, 42.1), n = 29
63 < dpi ≤ 93	26.7 (14.2, 44.4), n = 30	21.9 (11, 38.8), n = 32	26.1 (12.5, 46.5), n = 23	7.4 (2.1, 23.4), n = 27
Oral fluid	44.3 (35.8, 53.1), n = 122	33 (25.1, 42.1), n = 115	34.6 (27.6, 42.3), n = 156	28.8 (22.3, 36.2), n = 160
0 < dpi ≤ 7	83.3 (60.8, 94.2), n = 18 ^b^	20 (7, 45.2), n = 15	76.5 (52.7, 90.4), n = 17 ^b^	57.9 (36.3, 76.9) ^b^
7 < dpi ≤ 21	48.1 (30.7, 66), n = 27	50 (31.4, 68.6), n = 24	51.7 (34.4, 68.6), n = 29	41.4 (25.5, 59.3), n = 29 ^a^
21 < dpi ≤ 42	37.5 (22.9, 54.7), n = 32	37.5 (22.9, 54.7), n = 32	39.6 (27, 53.7), n = 48	35.4 (23.4, 49.6), n = 48
42 < dpi ≤ 63	7.7 (1.4, 33.3), n = 13	23.1 (8.2, 50.3), n = 13	3.3 (1, 16.7), n = 30 ^a^	9.4 (3.2, 24.2), n = 32
63 < dpi ≤ 93	40.6 (25.5, 57.7), n = 32	25.8 (13.7, 43.2), n = 31	18.8 (8.9, 35.3), n = 32	9.4 (3.2, 24.2), n = 32
Buccal swab	32.3 (25.5, 39.9), n = 158 ^a^	28.9 (22.4, 36.4), n = 159 ^a^	22 (16.2, 29.1), n = 159 ^a^	19.4 (14, 26.2), n = 160 ^a^
0 < dpi ≤ 7	36.8 (19.1, 59), n = 19 ^b^	15.8 (5.5, 37.6), n = 19	15.8 (5.5, 37.6), n = 19 ^b^	10.5 (2.9, 31.4), n = 19 ^b^
7 < dpi ≤ 21	35.7 (20.7, 54.2), n = 28	42.9 (26.5, 60.9), n = 28 ^a^	28.6 (15.3, 47.1), n = 28 ^a^	24.1 (12.2, 42.1), n = 29 ^a^
21 < dpi ≤ 42	43.8 (30.7, 57.7), n = 48	25 (14.9, 38.8), n = 48 ^d^	31.3 (19.9, 45.3), n = 48	22.9 (13.3, 36.5), n = 48 ^a^
42 < dpi ≤ 63	18.8 (8.9, 35.3), n = 32	43.8 (28.2, 60.7), n = 32 ^c^	18.8 (8.9, 35.3), n = 32	28.1 (15.6, 45.4), n = 32
63 < dpi ≤ 93	22.6 (11.4, 39.8), n = 31	15.6 (6.9, 31.8), n = 32	9.4 (3.2, 24.2), n = 32	6.3 (1.7, 20.1), n = 32
Tonsillar scraping	37.6 (30.4, 45.4), n = 157	27.8 (21.4, 35.3), n = 158 ^a^	21.9 (16.1, 28.9), n = 160 ^a^	25.3 (19.2, 32.7), n = 158 ^a^
0 < dpi ≤ 7	27.8 (12.5, 50.9), n = 18 ^b^	0 (0, 16.8), n = 19 ^a^	15 (5.2, 36), n = 20 ^b^	15.8 (5.5, 37.6), n = 19
7 < dpi ≤ 21	41.4 (25.5, 59.3), n = 29	39.3 (23.6, 57.6), n = 28 ^a^	34.5 (19.9, 52.7), n = 29 ^a^	34.5 (19.9, 52.7), n = 29 ^a^
21 < dpi ≤ 42	51.1 (37.2, 64.7), n = 47	56.3 (42.2, 69.3), n = 48 ^d^	36.2 (24, 50.5), n = 47	42.6 (29.5, 56.7), n = 47
42 < dpi ≤ 63	21.9 (11, 38.8), n = 32	12.5 (5, 28.1), n = 32 ^c^	6.3 (1.7, 20.1), n = 32 ^a^	9.4 (3.2, 24.2), n = 32
63 < dpi ≤ 93	35.5 (21.1, 53.1), n = 31	6.5 (1.8, 20.7), n = 31	9.4 (3.2, 24.2), n = 32	12.9 (5.1, 28.9), n = 31
**GROUP 2**	% (95% Confidence Interval), n		% (95% Confidence Interval), n	
Blood	35.7 (28.2, 43.9), n = 140 ^a^	36.4 (28.9, 44.5), n = 143 ^a^	29.4 (22.4, 37.6), n = 136 ^a^	34.8 (27.3, 43.1), n = 138 ^a^
0 < dpi ≤ 7	42.1 (23.1, 63.7), n = 19	31.6 (15.4, 54), n = 19	31.6 (15.4, 54), n = 19	42.1 (23.1, 63.7), n = 19
7 < dpi ≤ 21	51.7 (34.4, 68.6), n = 29	60.7 (42.4, 76.4), n = 28 ^a^	55.2 (37.5, 71.6), n = 29 ^a^	50 (32.6, 67.4), n = 28 ^a^
21 < dpi ≤ 42	41.7 (28.8, 55.7), n = 48	35.4 (23.4, 49.6), n = 48	23.8 (13.5, 38.5), n = 42	34.8 (22.7, 49.2), n = 46 ^a^
42 < dpi ≤ 63	22.7 (10.1, 43.4), n = 22	29.2 (14.9, 49.2), n = 24	16.7 (6.7, 35.9), n = 24	19 (7.7, 40), n = 21
63 < dpi ≤ 93	9.1 (2.5, 27.8), n = 22	20.9 (9.2, 40.5), n = 24	18.2 (7.3, 38.9), n = 22	25 (12, 44.9), n = 24
Oral fluid	33 (24.3, 43), n = 94	26.8 (19, 36.4), n = 97	20.1 (14.3, 27.6), n = 139	24.6 (18.3, 32.4), n = 142 ^b^
0 < dpi ≤ 7	36.8 (19.1, 59), n = 19	26.3 (11.8, 48.8), n = 19	26.3 (11.8, 48.8), n = 19	31.6 (15.4, 54), n = 19
7 < dpi ≤ 21	34.5 (19.9, 52.6), n = 29	27.6 (14.7, 45.7), n = 29	27.6 (14.7, 45.7), n = 29	27.6 (14.7, 45.7), n = 29
21 < dpi ≤ 42	37.5 (13.7, 69.4), n = 8	55.6 (26.7, 81.1), n = 9	23.4 (13.6, 37.2), n = 47	33.3 (21.7, 47.5), n = 48
42 < dpi ≤ 63	6.3 (1.1, 28.3), n = 16	25 (10.2, 49.5), n = 16	5 (1, 23.6), n = 20	9.1 (2.5, 27.8), n = 22
63 < dpi ≤ 93	45.5 (26.9, 65.3), n = 22 ^b^	16.7 (6.7, 35.9), n = 24	12.5 (4.3, 31), n = 24	12.5 (4.3, 31), n = 24
Buccal swab	17.3 (11.8, 24.5), n = 139 ^a^	17.5 (12.1, 24.6), n = 143 ^a^	9.1 (5.3, 15), n = 143 ^a^	9 (5.2, 14.9), n = 144 ^a,b^
0 < dpi ≤ 7	5.6 (1, 25.8), n = 18	10.5 (2.9, 31.4), n = 19	5.3 (1, 24.6), n = 19	15.8 (5.5, 37.6), n = 19
7 < dpi ≤ 21	24.1 (12.2, 42.1), n = 29	27.6 (14.7, 45.7), n = 29	13.8 (5.5, 30.6), n = 29 ^a^	13.8 (5.5, 30.6), n = 29 ^a^
21 < dpi ≤ 42	25 (14.9, 38.8), n = 48	16.7 (8.7, 29.6), n = 48	14.6 (7.2, 27.2), n = 48	10.4 (4.5, 22.2), n = 48 ^a^
42 < dpi ≤ 63	8.7 (2.4, 26.8), n = 23	12.5 (4.3, 31), n = 24	4.2 (1, 20.2), n = 24	0 (0, 13.8), n = 24
63 < dpi ≤ 93	9.5 (2.7, 28.9), n = 21	17.4 (7, 37.1), n = 23	0 (0, 14.3), n = 23	4.2 (1, 20.2), n = 24
Tonsillar scraping	22.3 (16.1, 30), n = 139	22.9 (16.8, 30.5), n = 144	14 (9.2, 20.7), n = 143 ^a^	15.5 (10.4, 22.4), n = 142 ^a^
0 < dpi ≤ 7	15.8 (5.5, 37.6), n = 19	36.8 (19.1, 59), n = 19	5.3 (1, 24.6), n = 19	36.8 (19.1, 59), n = 19
7 < dpi ≤ 21	32.1 (16.9, 50.7), n = 28	20.7 (9.8, 38.4), n = 29 ^a^	10.3 (3.6, 26.4), n = 29 ^a^	10.7 (3.7, 27.2), n = 28 ^a^
21 < dpi ≤ 42	27.7 (16.9, 41.8), n = 47	31.3 (19.9, 45.3), n = 48	25 (14.9, 38.8), n = 48	22.9 (13.3, 36.5), n = 48
42 < dpi ≤ 63	19 (7.7, 40), n = 21	12.5 (4.3, 31), n = 24	8.3 (2.3, 25.8), n = 24	0 (0, 13.8), n = 24
63 < dpi ≤ 93	8.3 (2.3, 25.8), n = 24 ^b^	8.3 (2.3, 35.8), n = 24	8.7 (2.4, 26.8), n = 23	4.2 (1, 20.2), n = 24

^a^ is a comparison to blood, ^b^ is a comparison to oral fluid, ^c^ is a comparison to buccal swabs, and ^d^ is a comparison to tonsillar scrapings within a column.

**Table 3:** Summary of agreement of results and correlation of cycle threshold values between two commercially available real-time PCR assays stratified by group, extraction, and sample type.

	Kappa Statistic	Correlation of Cycle Threshold Value	Positive Sample Cycle Threshold IDEXX	Positive Sample Cycle Threshold IDVet
	*Kappa (95% Conf. Interval)*		*Median (Range)*	*Median (Range)*
**GROUP 1**				
**MagMax Core Extraction**				
Blood	48.6 (33.9, 63.4)	0.78	37.1 (30.1, 39.4)	35.2 (27.5, 39.3)
Oral fluid	49.6 (33.8, 65.4)	−0.2 *	38.1 (35.5, 39.8)	36.9 (31.1, 39.7)
Buccal swab	43.9 (30, 57.8)	0.81	37.8 (31.2, 39.8)	37.2 (30.3, 39.8)
Tonsillar scraping	54.6 (41.5, 67.7)	0.64	37.3 (31, 39.9)	36.6 (31.0, 38.5)
**IDEXX Extraction**				
Blood	66.9 (54, 79.8)	0.83	36.4 (28.6, 39.7)	34.8 (28.9, 39)
Oral fluid	40.5 (23.6, 57.4)	−0.1 *	38 (33.8, 39.9)	37.1 (28.3, 39)
Buccal swab	55.7 (42.9, 68.4)	0.87	37.8 (31.7, 39.8)	37 (30.5, 39.2)
Tonsillar scraping	57.3 (44.6, 70.1)	0.67	37.1 (30.4, 39.8)	37 (30.6, 39.3)
**GROUP 2**				
**MagMax Core Extraction**				
Blood	64.4 (51.5, 77.3)	0.72	36.2 (31, 39.5)	34.9 (27.6, 38.1)
Oral fluid	56.4 (39.7, 73)	0.52 ^+^	38.2 (34.1, 39.8)	37.1 (32.4, 39.8)
Buccal swab	68.8 (57.1, 80.6)	0.83	37.2 (29.9, 39.9)	36.4 (31.7, 38.3)
Tonsillar scraping	62.1 (49.4, 74.8)	0.91	37.1 (28.4, 38.7)	36.4 (28.1, 38.9)
**IDEXX Extraction**				
Blood	61 (48.3, 73.8)	0.86	37 (31.7, 39.4)	35.9 (29.6, 39.1)
Oral fluid	62.2 (46.1, 78.2)	0.495 ^+^	38 (33.2, 39.6)	36.9 (32.8, 39.4)
Buccal swab	67.3 (55.6, 79)	0.86	38.2 (33.4, 39.3)	37 (33, 38.1)
Tonsillar scraping	65.8 (53.9, 77.7)	0.95	38 (28.7, 39.8)	37.4 (28.3, 39.2)

* denotes insignificant correlation coefficients at a 0.05 level of significance. ^+^ denotes significance at a level of significance of 0.1.

**Table 4:** Detection of antibodies against the MK-200 attenuated genotype 5 African swine fever virus variant stratified by group, days post infection, and diagnostic assay.

	IDVet	Innoceleris	Ingenasa
	% (95% Confidence Interval)	% (95% Confidence Interval)	% (95% Confidence Interval)
**GROUP 1**	n = 152	n = 96	n = 152
Serum, day ≥ 7	84.2 (22.5, 89.2)	84.4 (75.7, 90.4)	84.2 (22.5, 89.2)
Oral fluid, day ≥ 7	61.2 (53.2, 68.6)	46.9 (37.2, 56.8)	0.7 (0, 4)
	n = 144	n = 88	n = 144
Serum, day ≥ 12	88.9 (82.6, 93.1)	90.9 (82.8, 95.5)	88.9 (82.6, 93.1)
Oral fluid, day ≥ 12	68.4 (60.1, 75.6)	51.1 (40.9, 61.3)	0.7 (0, 4.2)
	n = 136	n = 88	n = 136
Serum, day ≥ 17	90.4 (84.2, 94.4)	90.9 (82.8, 95.5)	91.1 (85.1, 95)
Oral fluid, day ≥ 17	68.4 (60.1, 75.6)	51.1 (40.9, 61.3)	0.7 (0, 4.5)
**GROUP 2**	n = 136	n = 48	n = 136
Serum, day ≥ 7	75 (67.1, 81.6)	75 (61.1, 85.2)	69.1 (60.9, 76.3)
Oral fluid, day ≥ 7	64.6 (56.5, 71.9)	43.8 (30.7, 57.7)	0 (0, 3.3)
	n = 128	n = 48	n = 128
Serum, day ≥ 12	78.1 (70.2, 84.5)	75 (61.1, 85.2)	71.9 (63.5, 79)
Oral fluid, day ≥ 12	68.4 (60.1, 75.6)	51.1 (40.9, 61.3)	0 (0, 3.7)
	n = 120	n = 40	n = 120
Serum, day ≥ 17	80 (71.9, 86.2)	82.5 (68.1, 91.3)	74.2 (65.6, 81.2)
Oral fluid, day ≥ 17	65 (56.1, 73)	50 (35.2, 64.8)	0 (0, 3.7)

Innovative Diagnostics (IDVet) provided the IDScreen competitive ELISA for detection in serum and the IDScreen indirect ELISA for oral fluids. Innoceleris provided a single test assay with protocols for instruction on the separate matrices. Ingenasa’s assay was purchased, and the protocol only provides instructions for serum.

**Table 5:** Summary of agreement of results between commercially available antibody ELISA assays stratified by group, and sample type.

	Kappa Agreement Overall	Agreement between IDVet & Ingenasa	Agreement between IDVet & Innoceleris	Agreement between Ingenasa & Innoceleris
**GROUP 1**	% (95% Conf. Interval)	% (95% Conf. Interval)	% (95% Conf. Interval)	% (95% Conf. Interval)
Serum	79 (71.9, 86.1)	74.4 (66, 82.85)	95.7 (82.8, 100)	75.5 (61.2, 89.75)
Oral Fluid	34.1 (24.6, 43.6)	−4.55 (−19.45, 10.4)	75.5 (61.2, 89.75)	26.4 (11.5, 41.4)
**GROUP 2**				
Serum	61.3 (51.8, 70.8)	58.9 (48.7, 69.15)	89.3 (67.45, 100)	62.5 (40.6, 84.4
Oral Fluid	35.7 (25.2, 46.2)	3.6 (−11.7, 18.8)	78.6 (56.6, 100)	38.4 (17.2, 59.6)

## Data Availability

Not applicable.
